# Downregulation of the ubiquitin ligase KBTBD8 prevented epithelial ovarian cancer progression

**DOI:** 10.1186/s10020-020-00226-7

**Published:** 2020-10-27

**Authors:** Lei Du, Cong-Rong Li, Qi-Feng He, Xiao-Hua Li, Lin-Fei Yang, Yuan Zou, Zhi-Xia Yang, Dong Zhang, Xiao-Wei Xing

**Affiliations:** 1 Department of Center for Medical Experiments, The Third Xiang-Ya Hospital of Central South University, 138 Tongzipo Road, Changsha, 410013 Hunan China; 2 State Key Lab of Reproductive Medicine, Nanjing Medical University, 101 Longmian Ave., Nanjing, 211166 Jiangsu China; 3 The Affiliated Drum Tower Hospital of Nanjing University Medical School, 321 Zhongshan Road, Nanjing, 210008 Jiangsu China; 4 Hunan Provincial People’s Hospital, 90 Pingchuan Road, Changsha, 410205 Hunan China; 5 The Second Affiliated Hospital of Shanxi University of Traditional Chinese Medicine, 75 Jinci Road, Taiyuan, 030024 Shanxi China

**Keywords:** KBTBD8, Ubiquitin ligase, Epithelial ovarian cancer, Female fertility factor

## Abstract

**Objectives:**

Kelch repeat and BTB domain-containing protein 8, KBTBD8, has been identified as a female fertility factor. However, there have been no reports on the role of KBTBD8 in the progression of epithelial ovarian cancer, EOC. Our study aimed to address this issue.

**Methods:**

We first examine KBTBD8 expression in EOC tissues and cells. Next, we performed RNA sequencing to reveal the overall mechanism. Then we investigated the roles of KBTBD8 in the proliferation, migration, and health status of cultured EOC cells. Finally, we employed tumor xenograft models to evaluate the role of KBTBD8 in vivo.

**Results:**

First, KBTBD8 level was significantly higher in EOC tissues and cells. Next, comparative RNA sequencing identified more tumorigenesis-related genes that KBTBD8 might regulate. Then we found that KBTBD8 knockdown significantly decreased EOC cell proliferation, migration, and the activities of multiple tumorigenesis-related kinases. Finally, KBTBD8 knockdown significantly diminished ovarian tumor formation in vivo.

**Conclusion:**

Proper KBTBD8 level is essential for the healthy growth of ovarian somatic cells, such as ovarian epithelial cells. Excessive KBTBD8 might be a significant impetus for EOC progression. KBTBD8 reduction greatly inhibits EOC proliferation and migration.

## Background

Ovarian cancer is one of the most common malignant tumors of the female organs. Although the incidence of ovarian malignancy, mostly epithelial ovarian cancer (EOC), ranks third overall, the mortality rate in EOC patients is the highest among all types of gynecologic tumors (Bray et al. 2018; Smith et al. 2018; Torre et al. 2018; Chen et al. 2016; Matulonis et al. 2016). An unfortunate impediment to the successful treatment of patients with advanced ovarian cancer (AOC) is the inevitable drug resistance to conventional anticancer therapies (Pistollato et al. 2017; George et al. 2017).

The mechanism(s) governing ovary tumorigenesis is complex. Ubiquitination is an important type of protein post-translational modification that labels a target protein with mono- or polyubiquitin. Ubiquitination usually conduces to protein degradation by the proteosome complex. In this case, if ubiquitination is inhibited, the target protein will become hyperactivated; thus, the chance of tumorigenesis will increase. For example, the Notch signaling pathway is critically involved in breast and ovarian cancers, while the E3 ubiquitin protein ligase SCF complex is involved in NICD1 ubiquitination and degradation. Knockdown of FBXW7, a subunit of the SCF complex, promotes the migration and proliferation of breast cancer cells (Zhao et al. 2017). However, not all ubiquitination targets substrates for degradation. For example, the tumor suppressor proteins breast cancer type 1 (BRCA1) and BARD1 both contain an N-terminal zinc RING finger domain possessing E3 ubiquitin ligase activity; collectively they form a heterodimeric complex to maintain genome stability by ubiquitinating histone H2A at its C-terminal tail, which is essential for their optimal targeting to DNA damage sites and subsequent DNA repair (Shang et al. 2017; Densham et al. 2016; Mallery et al. 2002). Inherited missense mutations in the enzymatic RING finger domain in both BRCA1 (Kalb et al. 2014) and BARD1 (Serova et al. 1996) are highly correlated with the occurrence of ovarian cancer. Thus, it appears that different enzymes, even those possessing the same type of protein modification (e.g., ubiquitination), could have different functional mechanisms underlying EOC progression.

Kelch repeat and BTB domain-containing (KBTBD) protein family members are all composed of several kelch motifs and a BTB/POZ domain. The kelch motif mostly mediates ubiquitination (Stewart et al. 2018; Angers et al. 2006), while the BTB/POZ domain is involved in dimerization (Sun et al. 2009; Zipper and Mulcahy 2002). KBTBD family proteins are involved in ubiquitination in various cells and tissues and are correlated with human muscle diseases (Stewart et al. 2018; Angers et al. 2006; de la Luna et al. 1999; Gupta and Beggs 2014); however, none of the KBTBD members has been shown to be involved in EOC progression. In addition, KBTBD8 was the only KBTBD family member identified as a female fertility factor in a previous transcriptome-wide screen (Gallardo et al. 2007). And it was recently shown to be essential for oocyte quality by regulating the PKM1 level (Li et al. 2019). In the current study, we demonstrated for the first time that KBTBD8 is involved in EOC progression. Together, these results imply that a female fertility factor also plays important roles in EOC. Moreover, we also investigated the mechanism by which KBTBD8 functions.

## Materials and methods

### KBTBD8 immunohistochemistry of human ovarian cancer tissue microarray, imaging, and analysis

To investigate whether KBTBD8 protein level correlates positively with the degree of malignancy of human ovarian cancers, human ovary tissue microarray (BC11012b) containing 70 cases of ovarian cancer tissues, two cases of normal ovarian tissues, and one case of male adrenal gland tissue (as a tissue marker) were purchased from Xi’an Baisida Biotech Inc (Xi’an, Shanxi, China). The microarray was fixed, processed, and stained with KBTBD8 antibody according to the company’s immunohistochemistry instructions. For each array point, at least 500 randomly selected cells from five random areas were observed and analyzed by two independent professional analysts to generate a final average KBTBD8 staining score. The score for each array point was the accumulated value of the intensity score and proportion score. The legend for Additional file 1: Dataset 1 shows the detailed scoring rules.

### Cell lines and cell culture

The human normal ovarian epithelial cell line Moody, and two EOC lines, A2780 and HO8910, were purchased from the Cell Bank of the Chinese Academy of Sciences (Shanghai, China). All cells were cultured in Dulbecco’s modified Eagle’s medium (DMEM, Wisent, Montreal, Canada) supplemented with 10% fetal bovine serum (FBS, Fisher Scientific, Waltham, MA, USA) and 0.2 mM penicillin/streptomycin (Sigma, St. Louis, MO, USA) at 37 °C, 20% O_2_, and 5% CO_2_ in a humidified atmosphere. The scratch assay (wound healing) used DMEM with low FBS (1%).

### Western blotting

Harvested cells (1–2 million) were first lysed, then the gross proteins were separated by sodium dodecyl sulfate–polyacrylamide gel electrophoresis (SDS-PAGE) using an electroseparation device (Tanon, Shanghai, China). The proteins were then transferred onto a polyvinylidene fluoride (PVDF) membrane (Bio-Rad, Hercules, CA, USA) by an electro-transfer device (Tanon). Next, the membrane was incubated with primary and secondary antibodies in turn. Finally, the specific signals were amplified by an ECL (enhanced chemiluminescent) kit (Yeasen, Shanghai, China) and detected in an ECL detection device (Tanon).

### KBTBD8 shRNA transfection

Short hairpin RNA (shRNA) sequences (Additional file 2: Table 1) against the *KBTBD8* gene were designed and inserted into GV248 plasmid (GeneChem, Shanghai, China). The plasmids were purified with an endotoxin-free mini plasmid kit II (TIANGEN, Beijing, China). The cells were transfected with KBTBD8 or control plasmid using Lipofectamine 3000 Transfection Reagent (Fisher Scientific) according to the manufacturer’s instructions. After 24 h, the knockdown efficiency was verified by Western blotting. The cells were used for the subsequent in vitro or in vivo assays.

### *KBTBD8* overexpression

Full-length CDS (coding sequences) of *KBTBD8* and *EGFP* were cloned into pcDNA3.1 (+) by *Kpn*I/*Bam*HI (New England Biolabs, Ipswich, MA, USA) and BamHI/XbaI (New England Biolabs), respectively, to generate a KBTBD8-EGFP fusion expression vector. If required, a Flag sequence followed by a 6 × leucine sequence (to extend Flag outside for effective detection) was fused to the N-terminus of *KBTBD8*. This vector was used for *KBTBD8* overexpression (K8-OE) or *KBTBD8* knockdown-overexpression (K8-KD-OE) experiments through Lipofectamine 3000–mediated transfection. Additional file 2: Table 2 lists the detailed information for this construction.

### RNA sequencing

After centrifugation, 1 × 10^6^ control or KBTBD8-depleted cells were freshly frozen in liquid nitrogen and stored at − 80 °C. The samples were sent to BGI-Wuhan Company (Wuhan, Hubei, China) for RNA isolation, complementary DNA (cDNA) preparation, and DNA library preparation. The libraries were sequenced on an Illumina HiSeq.

### Quantitative PCR

mRNA reverse transcription was performed using a reverse transcription kit (Takara, Otsu, Japan). The cDNAs were amplified with a SYBR Green PCR Kit (Yeasen) on a CFX96 Real-Time PCR Detection System (Bio-Rad). Additional file 2: Table 3 shows the primer sequences used. Human β-actin RNA was amplified as the internal control. The levels of all other amplified gene fragments were shown as a ratio of the amplified signal/β-actin signal. The cDNA levels were calculated according to the comparative threshold cycle (2^−ΔΔCt^).

### Colony formation assay

Twenty-four hours after transfection, 200 cells were seeded onto each well of a 6-well plate and cultured for 6 days. The wells were then washed with phosphate-buffered saline (PBS), fixed with 1% paraformaldehyde, stained with 0.1% crystal violet, and observed under a light microscope (Nikon, Tokyo, Japan).

### Cell cycle analysis

After 24-h transfection and another 24-h culture, the harvested cells were fixed in 70% ice-cold ethanol and stored at 4 °C overnight. The fixed cells were washed twice with PBS, and then incubated with 100 μl propidium iodide (PI, 20 μg/ml) staining solution (Becton Dickinson, San Jose, CA, USA). The DNA content of the cells was qualified using a BD FACS Calibur flow cytometer (Becton Dickinson). The results were analyzed using FlowJo 2.8 software.

### Cell proliferation assay

The effect of *KBTBD8* knockdown on cell proliferation was detected by CCK-8 (Cell Counting Kit-8, Yi Fei Xue BioTechnology, Nanjing, Jiangsu, China). The cells (5 × 10^3^ cells/well in logarithmic growth phase) were first seeded into 96-well plates. Then, 10 μl CCK-8 solution was added to each well and incubated for 4 h. The absorbance, which is positively related to the number of proliferating cells, was measured at an OD (optical density) of 450 nm.

### Wound healing assay

After 24-h transfection, 5 × 10^5^ cells per well were grown to 80–90% confluence in 6-well plates. The monolayer was then neatly scratched using a sterile 10-μl pipette tip. Three photographs of randomly selected wound areas were taken within specific time points (0, 24, 48, 72 h) under a light microscope (Olympus Corp, Tokyo, Japan). The wound areas were analyzed by ImageJ software.

### Cell invasion assay

Matrigel invasion chambers (Jet Bio-Filtration, Guangzhou, Guangdong, China) were used for the cell invasion assay. Briefly, cells were seeded in the upper chamber with medium containing 0.1% bovine serum albumin (BSA), while medium containing 30% FBS was placed in the lower chamber. After 24-h incubation at 37 °C, invasive cells that had invaded to the bottom of the membrane were fixed with 1% formalin, stained with 0.1% crystal violet, and observed under a Nikon light microscope.

### Detection of ROS generation

Reactive oxygen species (ROS) were detected using an ROS Assay Kit (Beyotime, Shanghai, China). Cells were first seeded onto glass slides, then incubated with dichlorofluorescein diacetate probe for 20 min at 37 °C in darkness. Images were obtained with an Andor spinning disk confocal workstation (Oxford Instruments, Abingdon, UK).

### Assay of mitochondrial membrane potential

Cells were incubated at 37 °C for 20 min with JC-1 diluted at 1:200 (Yeasen), then the green fluorescent (JC-1 as a monomer at low membrane potentials) and the red fluorescent (JC-1 as J-aggregates at higher membrane potentials) images were captured as above. A decrease in the red/green fluorescence intensity ratio indicated mitochondrial depolarization.

### Detection of early apoptosis

Apoptosis was detected using an Annexin V-FITC/PI Apoptosis Detection Kit (Yeasen). Cells were first seeded onto glass slides, and then stained with 5 μl Annexin V-FITC (fluorescein isothiocyanate) and 10 μl PI Staining Solution for 15 min in darkness at room temperature. Then, images were obtained as above.

### Antibodies

Primary antibodies: mouse monoclonal anti-GAPDH (Cat# 30201ES60) was bought from Yeasen. Rabbit polyclonal anti-KBTBD8 antibody was produced by Zoonbio Biotechnology (Nanjing, Jiangsu, China). Phosphorylated (p)-ERK1/2 (Cat# M9692) and ERK1/2 (Cat# M5670) antibodies were from Sigma. P-RPS6 (Cat# BS4359), RPS6 (Cat# BS1592), and ubiquitin (A46) antibodies were from BioWorld (Bloomington, TX, USA). P-mTOR (Cat# AP0094) and mTOR (Cat# A2445) antibodies were from ABclonal (Wuhan, Hubei, China). P-Akt^S473^ (Cat# 4060) antibody was from Cell Signaling (Danvers, CO, USA). P-Src (Cat# D151209-0025) and Akt (Cat# D1516-0100) antibodies were from BBI Life Sciences (Shanghai, China). Src (Cat# 11097-1-AP) antibody was from Proteintech (Rosemont, IL, USA).

Secondary antibodies: Cy2-conjugated donkey anti-mouse immunoglobulin G (IgG) (Code: 715-225-150), rhodamine (TRITC)-conjugated donkey anti-human IgG (Code: 709-025-149), Cy2-conjugated donkey anti-human IgG (Code: 709-225-149), and Cy2-conjugated donkey anti-rabbit IgG (Code: 711-225-152) were from Jackson ImmunoResearch Laboratory (West Grove, PA, USA). Horseradish peroxidase (HRP)-conjugated rabbit anti-goat IgG and HRP-conjugated goat anti-mouse IgG were from Vazyme (Nanjing, Jiangsu, China).

### Co-immunoprecipitation (Co-IP)

Anti-CUL3 IgG, anti-KBTBD8 IgG, or control rabbit IgG (5 μg) were coupled to 30 μl protein A/G beads (Macgene, Beijing, China) for 4 h at 4 °C on a rotating wheel in 250 μl IP buffer (20 mM Tris–HCl, pH 8.0, 10 mM EDTA, 1 mM EGTA, 150 mM NaCl, 0.05% Triton X-100, 0.05% NP-40, 1 mM phenylmethylsulfonyl fluoride) with protease inhibitor (Sigma). Meanwhile, 2.5 × 10^6^ cells were lysed in 250 μl IP buffer, and the supernatant was pre-cleaned with 30 μl protein A/G beads for 4 h at 4 °C. After that, the IgG-coupled beads were incubated with pre-cleaned cell lysate supernatant overnight at 4 °C. Finally, the resulting beads with the bound immunoprecipitates were underwent Western blotting with antibodies against KBTBD8 or CUL3 in parallel.

### Nude mouse xenograft tumor model

All animal-related experiments were approved by the Committee on Animal Ethics of Nanjing Medical University (Nanjing, Jiangsu, China). *KBTBD8*-depleted A2780 cells or control cells (1 × 10^7^) were injected subcutaneously into the left upper flank of 4-week-old female immunodeficient nude mice. After 3 weeks, the mice were anesthetized and then sacrificed. Tumor diameters were measured with digital calipers, and the tumor volume in mm^3^ was calculated using the following formula: Volume = 0.5 × (Width) 2 × Length.

### TUNEL assay

Cells plated on coverslips were fixed with 10% paraformaldehyde for 10 min, permeabilized in 0.1% Triton X-100 for 5 min, and blocked in 1% BSA for 20 min. Next, the coverslips underwent TUNEL (terminal deoxynucleotidyl transferase-mediated dUTP nick end-labeling) assay according to the manufacturer’s instructions (Yeasen). DNA was stained with 10 ng/ml DAPI (4,6-diamino-2-phenylindole), and images were obtained as above.

### Quantification

Strict and consistent quantification principles was observed for all quantification. ImageJ was used for all quantification. For measuring band intensity in the Western blot experiments, the net intensity was first obtained from the measured intensity value, then the background intensity around the band was subtracted; next, the net integrated intensity value was obtained from the net intensity value multiplied by the band area. Finally, the normalized final band value was obtained from the net integrated intensity value divided by that of the loading control band (GAPDH, actin, or tubulin).

For measuring signal intensity in immunofluorescence, live dye staining, or immunohistochemistry, whole-cell net intensity was first obtained by subtracting the background intensity outside the cell from the measured intensity. Then, the normalized final value was obtained by the net integrated intensity value divided by the background intensity.

### Statistical analysis

Statistical analysis was performed using SPSS standard version 13.0. Data were from at least three independent repeats and are presented as the mean ± SEM. Comparisons between groups was determined by Student’s *t*-test or one-way analysis of variance (ANOVA). The relationship between KBTBD8 protein expression and the pathologic characteristics of the tissue microarrays was analyzed using the Kruskal–Wallis H test. P < 0.05 was considered statistically significant.

## Results

### Human KBTBD8 was overexpressed in multiple clinical ovarian cancer samples

Although a total of 13 KBTBD family members are currently known, none have been reported to be involved in EOC progression. There are several reasons why we chose to focus on KBTBD8. First, in a previous transcriptome-wide screen of genes required for follicle maturation and development, KBTBD8 was the only member of the KBTBD gene family that was identified as a female fertility factor (Gallardo et al. 2007). And it was recently shown to be essential for oocyte quality by regulating the PKM1 level (Li et al. 2019). Thus, we aimed to determine whether this female fertility factor is also important in EOC progression. Second, unlike other KBTBD members that are involved in ubiquitination-mediated protein degradation, ubiquitination by KBTBD8 stabilizes the substrates (Werner et al. 2015), thus its overexpression would presumably cause hyperstability and induce tumorigenesis.

**Fig. 1:**
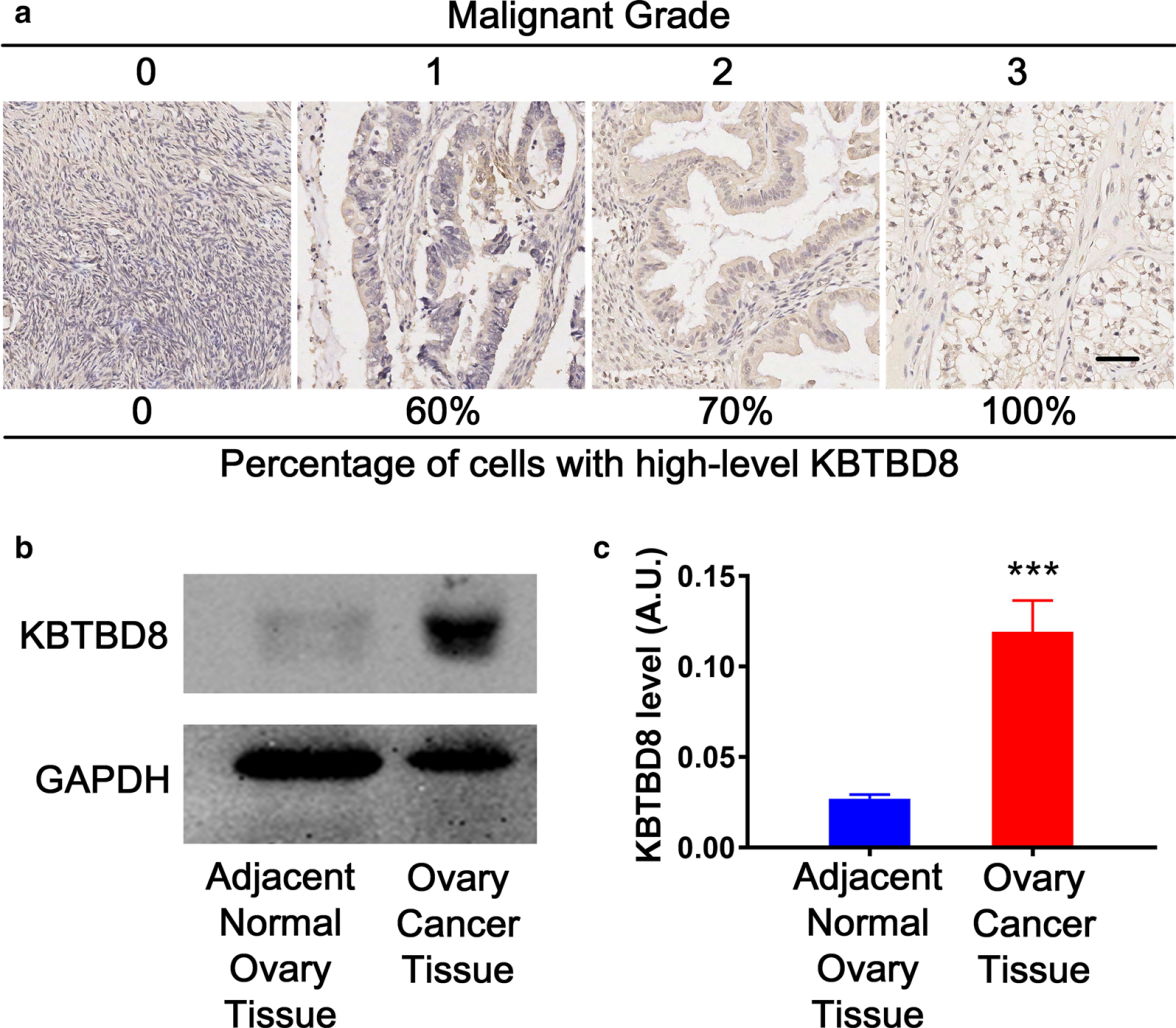
Human KBTBD8 was overexpressed in multiple clinical ovarian cancer samples. **a** KBTBD8 immunohistochemistry on an ovary tissue microarray showed that KBTBD8 level was highly correlated with the malignancy grade of ovarian cancer (Detailed information for each array point is in Additional file 1: Dataset 1). The malignancy grade and corresponding percentage of cells with high-level KBTBD8 are: 0 (0), 1 (60%), 2 (70%), and 3 (100%). **b**, **c** Western blot and quantification showed that the KBTBD8 protein level in ovarian cancer tissue was about fourfold higher than in adjacent normal ovarian tissue (Adjacent normal ovarian tissue vs. ovarian cancer tissue, 0.02689 vs. 0.1190). A.U., arbitrary unit. Scale bar, 100 μm. ***Indicates p < 0.001

We first evaluated KBTBD8 protein levels using immunohistochemistry on a human ovarian cancer tissue microarray and found that KBTBD8 levels were highly correlated with the malignancy grade of ovarian cancer (Fig. 1a, Table 1, Additional file 1: Dataset 1). Western blot analysis also showed that KBTBD8 protein levels in ovarian cancer tissue were approximately fourfold higher than that in normal ovarian tissue (Fig. 1b, c, to make sentences concise, we included all exact values into the figure legends). These results indicate that KBTBD8 might be involved in ovarian cancer progression.

**Table 1:** Correlation of KBTBD8 protein level and the malignance grade of the ovarian tissues

Feature	KBTBD8 staining score^a^			N^b^	*p* value^c^
	−	+	++		
Age(years)					
≤ 48	8	16	11	35	0.037
> 48	3	17	15	35	
TNM-stage					
–	4	2	6	12	0.03
TI	6	15	11	32	
TII	0	10	5	15	
TIII	1	6	4	11	
Lymph node metasitasis					
Yes	0	6	3	9	0.688
No	11	27	23	61	
Multifocality					
Yes	0	2	0	2	0.626
No	11	31	26	68	
Disease grade					
–	9	4	5	18	0.00
1	1	8	5	14	
2	1	11	3	15	
3	0	10	13	23	
TNM stage group					
–	4	2	6	12	0.021
I	6	15	11	32	
II	0	8	5	13	
III	1	6	4	11	
IV	0	2	0	2	
Percent of positive cells(PP)					
≤ 50	11	23	2	36	0.00
> 50	0	10	24	34	

This table was generalized from Additional file 1: Dataset 1. The intensity score of KBTBD8 staining of each array point was standardized as follows: 0, no staining; 1, weak staining; 2, moderate staining; and 3, strong staining. For each array point, at least 500 randomly-picked cells from five random areas were observed and analyzed by two independent professional analyzers to generate a final average score

The proportion score of KBTBD8-stained tumor cells was standardized as follows: 0, < 5% of cells with positive KBTBD8 staining; 1, 5–25%; 2, 25–50%; 3, 50–75%; and 4, > 75%

Next the final score was obtained by the intensity score added together with the proportion score. A final score of 0–1 was defined as (−), while scores of 1–2, 3–4, and 5–6 were considered (+), (++) and (+++), respectively

^a^The KBTBD8 antibody staining Score method is detailed as follow

^b^N stands for the number of cases

^c^p values were calculated by Kruskal–Wallis H test tests. A value of p < 0.05 was considered statistically significant

### Numerous cellular processes were affected by KBTBD8 knockdown, as assessed by RNA sequencing

**Fig. 2:**
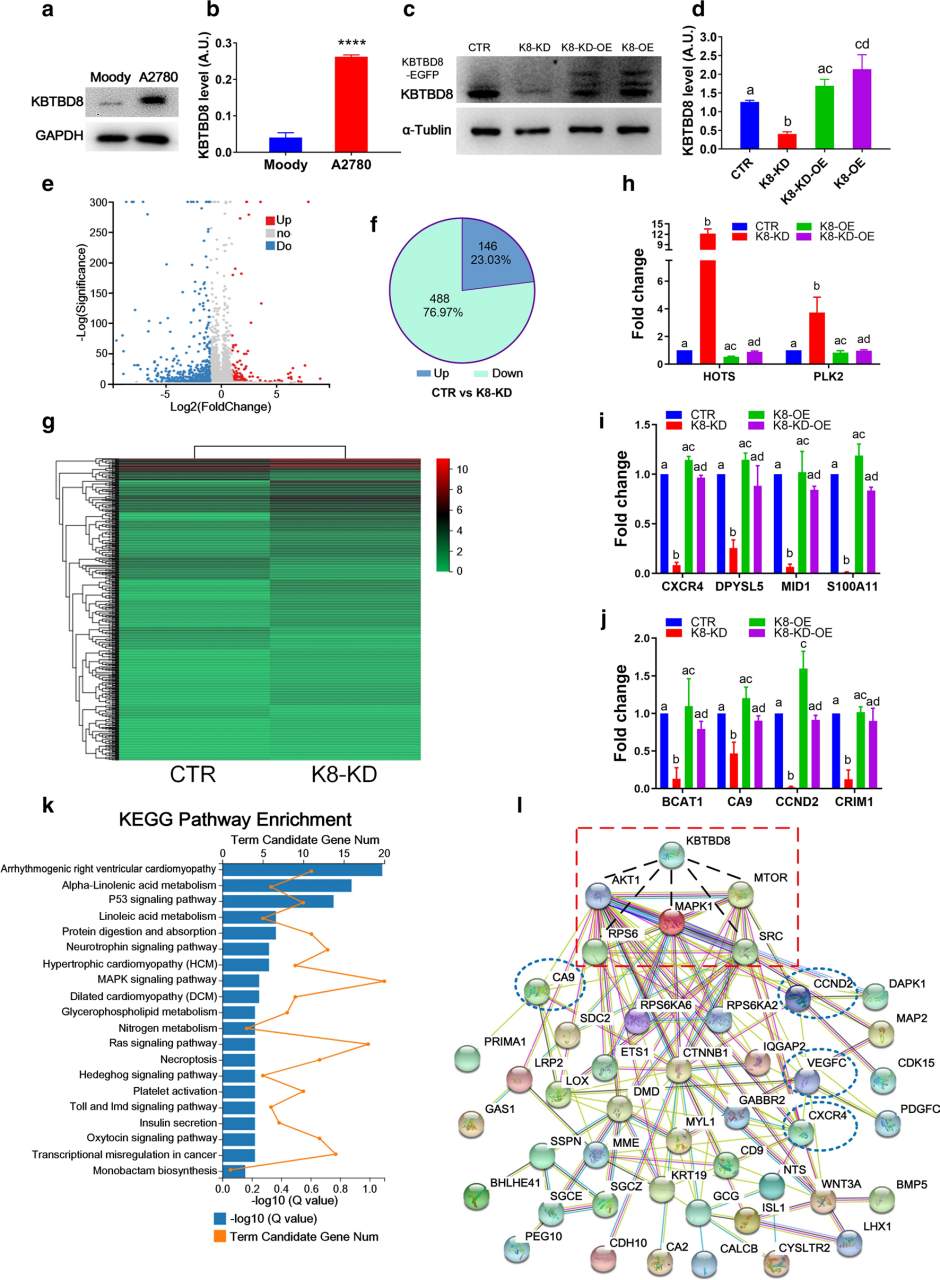
Numerous cellular processes were affected by KBTBD8, as assessed by RNA sequencing. **a**, **b** Blot and quantification showed that KBTBD8 protein level was about sixfold higher in A2780 EOC cells than in normal epithelial ovarian cell line moody (KBTBD8 protein level, moody vs. A2780, 0.04061 vs. 0.2623). **c**, **d** Blot and quantification showed that KBTBD8 protein level increased in KBTBD8-knockdown-overexpressed (K8-KD-OE) and KBTBD8-overexpressed (K8-OE) A2780 cells and decreased in KBTBD8-knockdown (K8-KD) A2780 cells compared with the control A2780 cells (CTR vs. K8-KD vs. K8-KD-OE vs. K8-OE, 1.259 vs. 0.4056 vs. 1.692 vs. 2.137). **e** Scattered map of upregulated (red) and downregulated (blue) genes which were positioned away from unchanged genes (gray). **f** Pie Chart of differentially expressed genes. 146 genes were significantly upregulated (|log2FC|≥ 1), and 448 genes were significantly downregulated (|log2FC|≥ 1). The downregulated genes cover 76.97% (448/594) of all differentially-expressed genes. **g** Heat map of differentially-expressed genes between CTR and KBTBD8-knockdown group. **h**–**j** Q-PCR of some screened genes verified the creditability of the sequencing, and K8-OE could rescue the changes caused by K8-KD. **k** KEGG analysis showed that many of the proteins that these genes encode are involved in multiple tumorigenesis pathways. **l** Protein interaction analysis by String showed that that 39 functionally essential proteins encoded by differentially expressed genes interact with multiple kinases that are known to be important for tumorigenesis. Different characters above the column between groups indicate significant difference. ***Indicates p < 0.001, ****indicates p < 0.0001

To fully investigate the mechanism by which KBTBD8 regulates EOC progression, we planned to first perform comparative RNA sequencing on control and KBTBD8-knockdown EOC tissues. But due to the complexity of its origin, it is very difficult to pick proper human tissues. Since EOC is the most common type of ovarian cancer and has the highest mortality rate among all types of gynecologic tumors (Bray et al. 2018; Smith et al. 2018), we decided to do RNA sequencing on immortalized EOC cell lines. We first determined that the KBTBD8 protein levels were about six or tenfold higher in A2780 (Fig. 2a and b) or HO8910 (Additional file 3: Fig. 1A and B) cells than in the normal ovarian epithelial cell line, Moody. Therefore, these two EOC cell lines were appropriate for in vitro EOC studies. To allow appropriately standardized comparisons between individual images, we included all results obtained from HO8910 cells in the Additional file 3: Figure sections. Next, we transfected cells with specific KBTBD8 shRNA-inserted GV248 plasmids (Additional file 2: Table 1) and determined that they were able to reduce KBTBD8 expression in both cell lines dramatically (Fig. 2c and d, Additional file 3: Fig. 1C and D). Next, RNA sequencing on control and KBTBD8-depleted A2780 cells demonstrated that 146 genes were significantly upregulated (|log2FC|≥ 1), while 448 genes were significantly downregulated (|log2FC|≥ 1) (Fig. 2e–g, Additional file 1: Datasets 2 and 3). The downregulated genes accounted for 75.4% (448/594) of all the differentially expressed genes, indicating that KBTBD8 functions primarily by stabilizing substrates (Fig. 2f).

Q-PCR analysis of some of the differentially expressed genes verified the creditability of the RNA sequencing results (Fig. 2h–j, Additional file 2: Table 3). To further verify this result, we also did KBTBD8 overexpression only (K8-OE) and KBTBD8 overexpression after knockdown (K8-KD-OE) (Fig. 2c, d, h–j). In sum, the alteration trends of mRNA levels in K8-OE group were contrary to K8-KD group compared with control group; And mRNA levels in K8-KD-OE group were close to control level (Fig. 2h–j), indicating that K8-OE could rescue the changes caused by K8-KD. Kyoto Encyclopedia of Genes and Genomes (KEGG) analysis showed that many of the proteins encoded by these genes are involved in multiple essential pathways, such as MAPK, Ras, oxytocin, hedgehog, Toll, and p53, which encompass proliferation, migration, and survival (Fig. 2k, Additional file 2: Table 4). A protein interaction analysis performed with the STRING database showed that 39 functionally essential proteins encoded by differentially expressed genes (Additional file 1: Dataset 4) interact with multiple kinases that are known to be important for cell proliferation, migration, and differentiation (Fig. 2l, surrounded by red dotted lines). These results suggested that the KBTBD8 level might be important for EOC progression through regulating genes within multiple cancer-related pathways.

### Human KBTBD8 is a critical factor for the proliferation and migration of EOC cells

Based on the results described above, it appears that KBTBD8 might be involved in ovarian tumorigenesis, we next investigate the involvement of KBTBD8 in EOC progression in vitro.

**Fig. 3:**
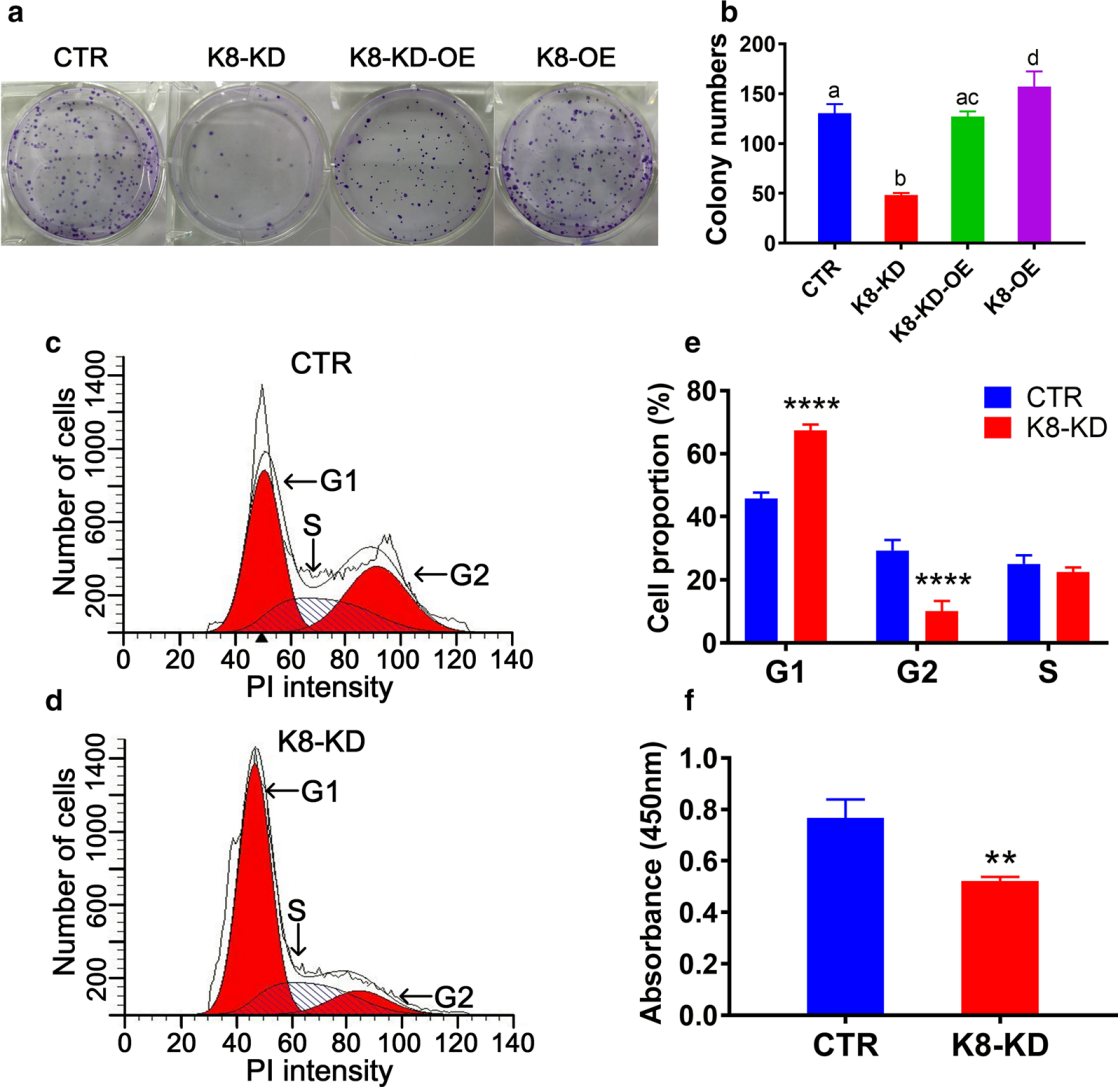
Human KBTBD8 was important for the proliferation of EOC cells. **a**, **b** The colony number in K8-OE group was significantly more than control group, whereas the colony number in K8-KD-OE group was more than K8-KD group while close to control group (Colony number, CTR vs. K8-KD vs. K8-KD-OE vs. K8-OE, 130 vs. 48 vs. 127 vs. 157). **c**–**e** Cell cycle analysis by FACS showed that significantly fewer cells were at the G2 stage (proportion of cells at G2, CTR vs. K8-KD, 29.22% vs 10.09%) and more cells were at the G1 stage (percentage of cells at G1, CTR vs. K8-KD, 45.72% vs. 67.39%) in KBTBD8-knockdown A2780 cells than in control cells. PI, Propidium Iodide. **f** CCK8 assay showed that there were less proliferating cells in the KBTBD8-knockdown A2780 cells than in control cells (CCK8, CTR vs. K8-KD, 0.7672 vs. 0.5211). A.U., arbitrary unit. Different characters above the column between groups indicate significant difference. **Indicates p < 0.01, ****indicates p < 0.0001

We used colony formation assay to assess cell proliferation. Results showed that the numbers of colonies formed after 7 days were significantly decreased by twofold in KBTBD8-knockdown (K8-KD) A2780 (Fig. 3a and b) or HO8910 (Additional file 3: Fig. 1e and f) cells compared with the numbers in control cells. To further verify this result, we also set up K8-OE and K8-KD-OE groups (Fig. 2c and d). We found that the colony number in K8-OE group was significantly more than control group, whereas the colony number in K8-KD-OE group was more than K8-KD group while close to control group (Fig. 3a and b), indicating that K8-OE could rescue the changes caused by K8-KD. Next, cell cycle analysis by FACS showed considerably more cells in G1 phase (interphase) in the K8-KD A2780 (Fig. 3c–e) or HO8910 (Additional file 3: Fig. 1G–I) cells than in control cells. Finally, CCK8 assay revealed fewer proliferating cells in the K8-KD A2780 cells (Fig. 3f) or HO8910 cells (Additional file 3: Fig. 1J) than in control cells. These results indicate that high levels of KBTBD8 promote the proliferation of EOC cells.

**Fig. 4:**
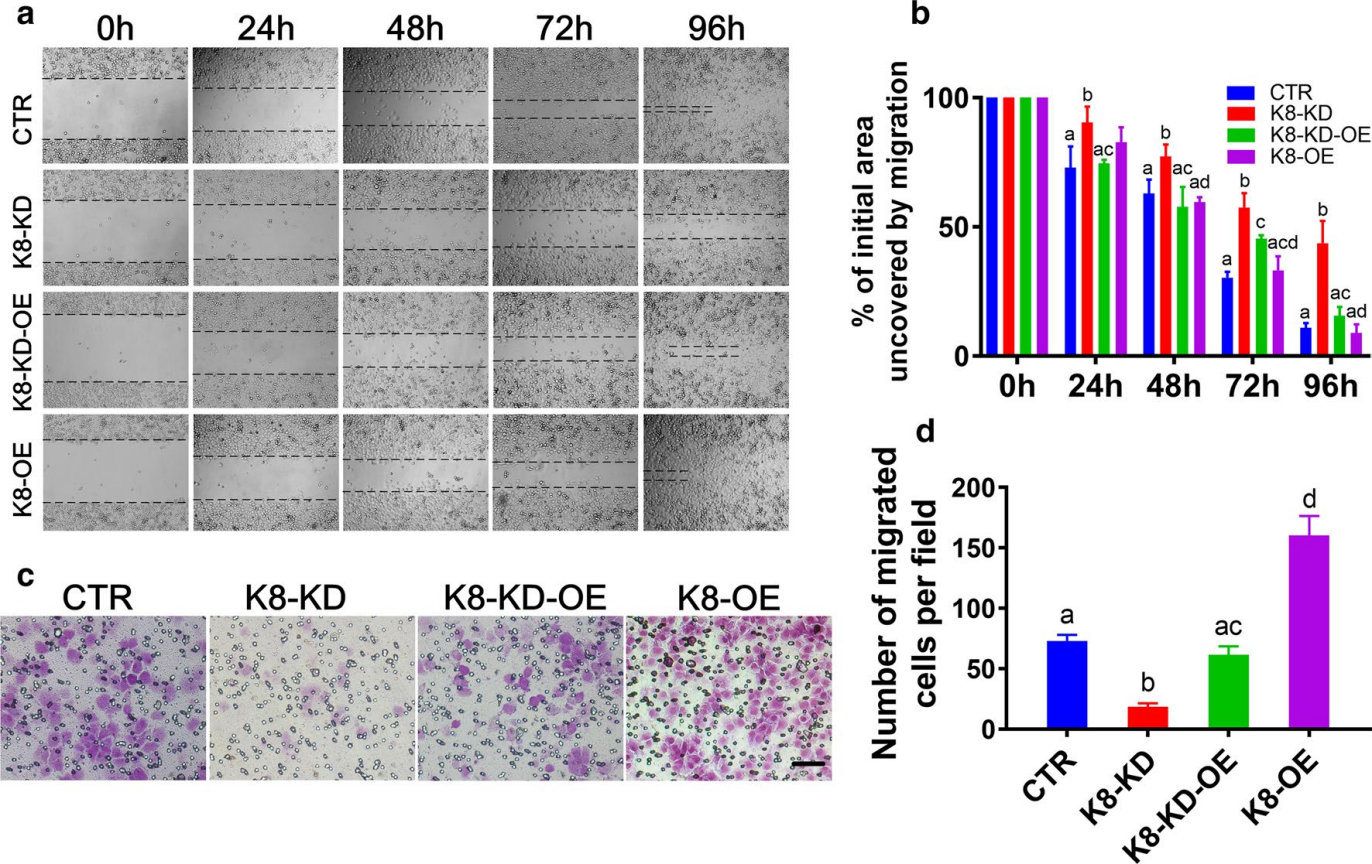
Human KBTBD8 was important for the migration of EOC cells. **a**, **b** Wound healing assays showed that the closure ratio in K8-OE group was more than in control group; whereas the closure ratio in K8-KD-OE group was more than K8-KD group while close to control group. Representative images at 0 h, 24 h, 48 h, 72 h, and 96 h were shown. Percentage of initial scratched area uncovered by migrated cells at 24 h, CTR vs. K8-KD vs. K8-KD-OE vs. K8-OE, 72.99% vs. 90.38% vs. 74.58% vs. 82.79%; at 48 h, 62.95% vs. 77.26% vs. 57.82% vs. 59.50%; at 72 h, 30.29% vs. 57.35% vs. 45.35% vs. 33.13%; at 96 h, 10.90% vs. 43.61% vs. 15.54% vs. 8.896%. **c**, **d** 3-dimensional migration assay showed that the number of migrated cells in K8-OE group was more than in control group, whereas the number of migrated cells in K8-KD-OE group was more than in K8-KD group while close to control group (Number of migrated cells per field, CTR vs. K8-KD vs. K8-KD-OE vs. K8-OE, 73 vs. 19 vs. 62 vs. 160). Scale bar, 50 μm. Different characters above the column between groups indicate significant difference

We used wound healing assay to assess cell migration. Results showed that the closure ratio of the scratched area was significantly slower in the K8-KD (Fig. 4a and b) or HO8910 (Additional file 3: Fig. 2A and B) cells than in control cells. Moreover, the closure ratio in K8-OE group was more than in control group; whereas the closure ratio in K8-KD-OE group was more than K8-KD group while close to control group (Fig. 4a and b). We then used transwells to test cellular migration in 3 dimensions. We found that the number of migrated cells significantly decreased by four to ninefold in the K8-KD A2780 (Fig. 4c and d) or HO8910 (Additional file 3: Fig. 2C and D) cells relative to the control cells. Moreover, the number of migrated cells in K8-OE group was more than in control group, whereas the number of migrated cells in K8-KD-OE group was more than in K8-KD group while close to control group (Fig. 4c and d). These results indicate that high levels of KBTBD8 promote the migration of EOC cells.

These results suggest that high levels of KBTBD8 promote the proliferation and migration of EOC cells.

### Human KBTBD8 is important for the overall health status of EOC cells

**Fig. 5:**
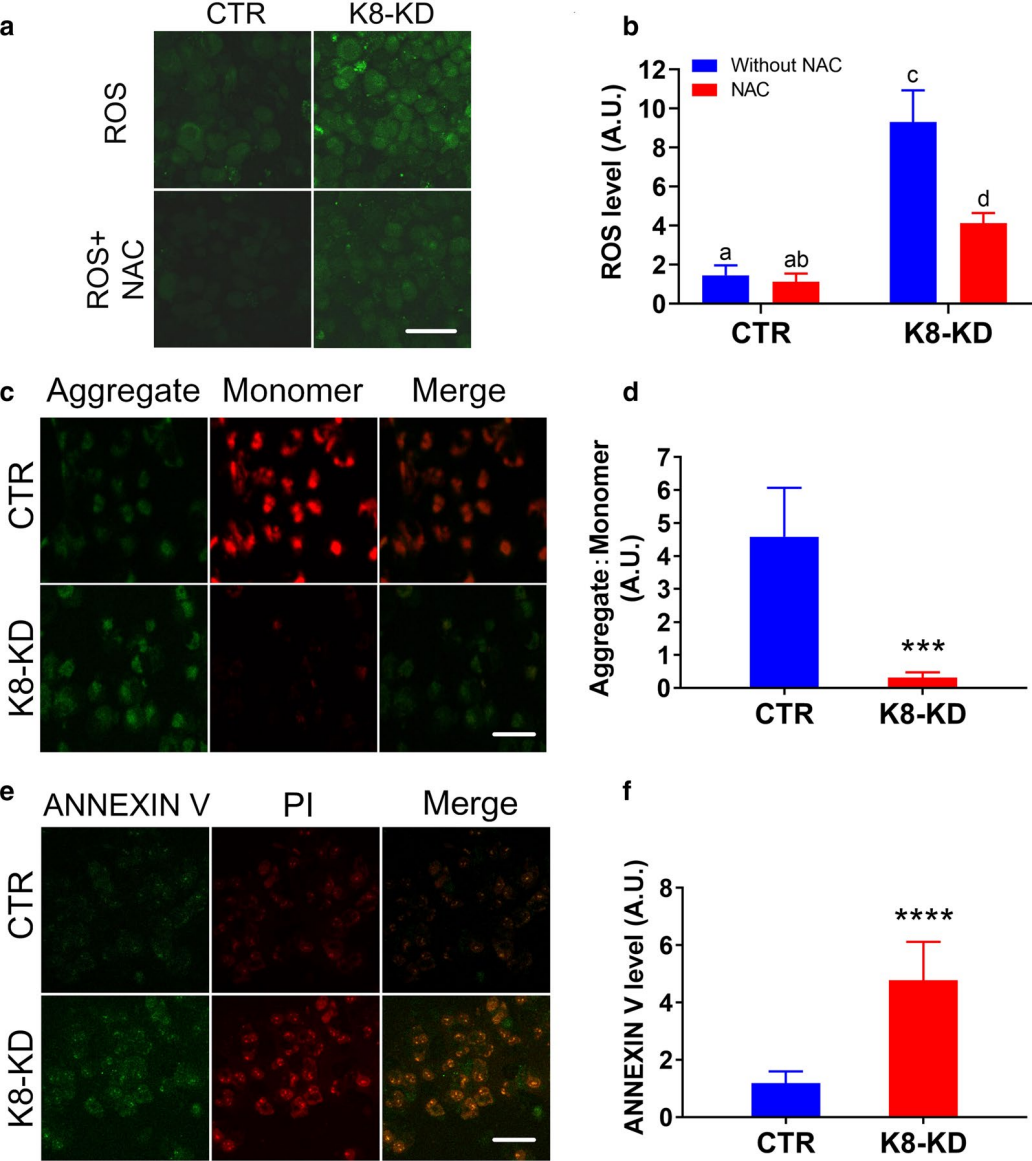
Human KBTBD8 was important for the general health state of EOC cells. **a**, **b** KBTBD8 knockdown significantly increased ROS level in A2780 ovarian cancer cells with or without NAC (ROS level without NAC, CTR vs. K8-KD, 1.458 vs. 9.313; ROS level with NAC, CTR vs. K8-KD, 1.125 vs. 4.125). **c**, **d** JC-1 staining assay showed that the mitochondria membrane potential significantly decreased after the KBTBD8 knockdown (Aggregate: monomer, CTR vs. K8-KD, 4.580 vs. 0.3118). Aggregate in green, monomer in red. **e**, **f** Annexin V staining assay showed that KBTBD8 knockdown significantly increased the apoptosis level (Annexin V level, CTR vs. K8-KD, 1.183 vs. 4.775). Annexin V signal in green, PI in red. A.U., arbitrary unit. Scale bar, 50 μm. Different characters above the column between groups indicate significant difference. ***Indicates p < 0.01, ****indicates p < 0.0001

Since immortality is another key feature of cancer cells, we performed several classical assays to assess the health status of EOC cells after KBTBD8 knockdown. First, with or without NAC treatment (ROS scavenger), KBTBD8 knockdown significantly increased ROS levels by four or fivefold in A2780 (Fig. 5a and b) or HO8910 (Additional file 3: Fig. 3A and B) cells. Second, the JC-1 assay revealed that the mitochondrial membrane potential was significantly reduced by 14 or tenfold in A2780 (Fig. 5c and d) or HO8910 (Additional file 3: Fig. 3C and D) cells after KBTBD8 knockdown. Finally, by annexin V staining, we found that KBTBD8 knockdown significantly increased apoptosis by four or threefold in A2780 (Fig. 5e and f) or HO8910 (Additional file 3: Fig. 3E and F) cells.

ROS are toxic byproducts of aerobic metabolism, a high level of ROS indicates that aerobic metabolism is abnormally high. The mitochondrial membrane potential is a crucial marker for mitochondrial integrity. A significantly decreased potential indicates severe dysfunction in mitochondria. A high level of annexin V indicates an increased chance of apoptosis. These results suggest that KBTBD8 knockdown impairs cancer cell immortality by impairing the overall health status via multiple vital aspects.

### Human KBTBD8 knockdown inactivated multiple cell cycle kinases in EOC cells

**Fig. 6:**
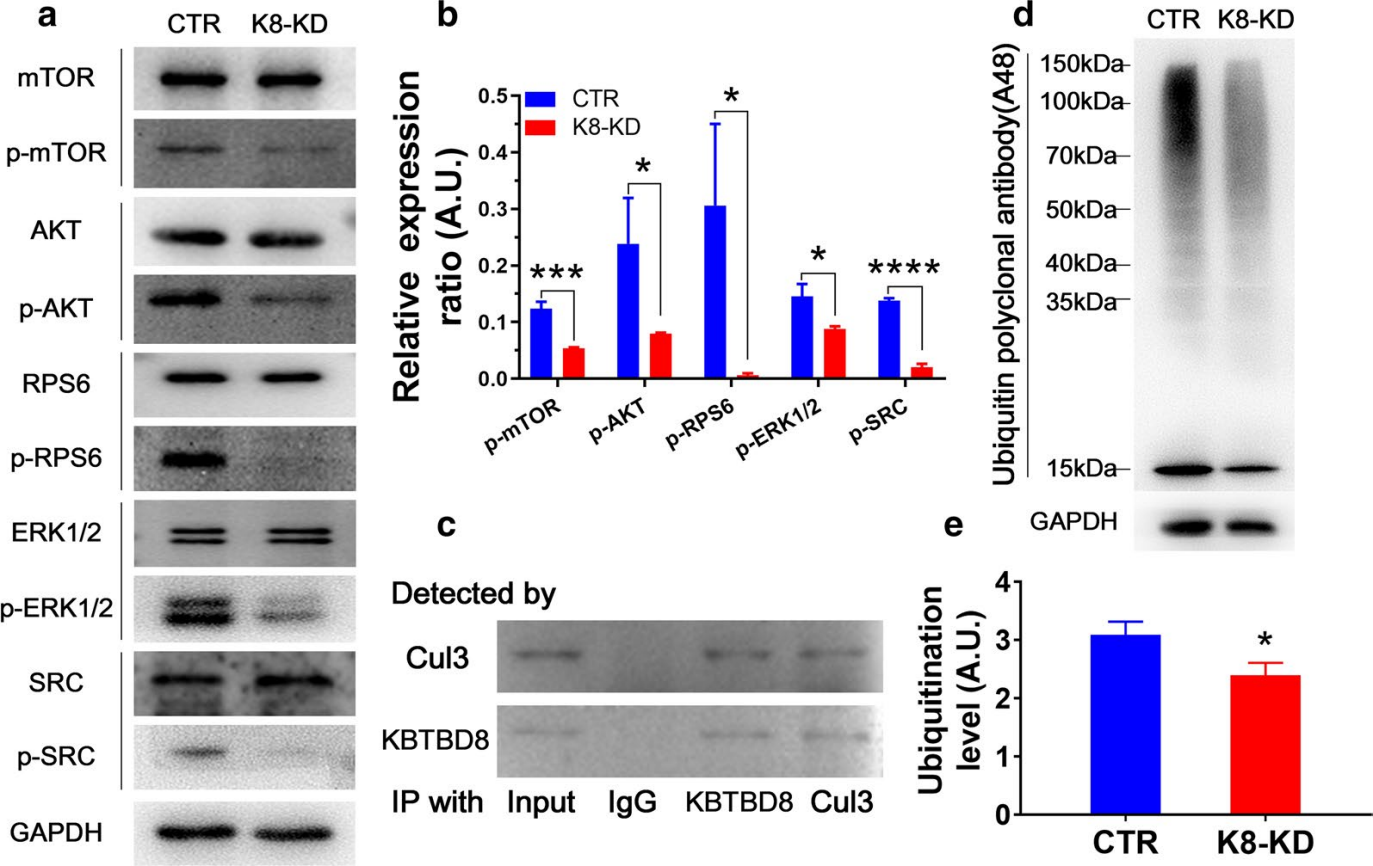
Human KBTBD8 regulated the activity of multiple cell cycle kinases in EOC cells. **a**, **b** Blot and quantification showed that KBTBD8 knockdown decreased the level of p-MTOR, p-Akt, p-Rps6, p-Erk1/2 and p-Src in A2780 cells (CTR vs. K8-KD, p-MTOR level, 0.1240 vs. 0.05371; p-Akt level, 0.2387 vs. 0.07942; p-Rps6 level, 0.3059 vs. 0.006376; p-Erk1/2 level, 0.1456 vs. 0.08771; p-Src level, 0.1378 vs. 0.02054), while KBTBD8 knockdown did not decreased the level of total MTOR, Akt, Rps6, Erk1/2, and Src level in A2780 cells. **c** Co-IP and blot showed that cul3 and KBTBD8 interacted with each other. **d**, **e** Blot and quantification showed that KBTBD8 knockdown significantly diminished the overall level of ubiquitination (ubiquitination level, CTR vs. K8-KD, 3.085 vs. 2.391). A.U., arbitrary unit. *Indicates p < 0.05, ***indicates p < 0.001, ****indicates p < 0.0001

From the RNA sequencing results, 39 proteins encoded by the DEGs between control and K8-KD group closely interact with multiple kinases known to be important for tumorigenesis. Therefore, we next examined whether KBTBD8 regulates the activities of these kinases. KBTBD8 knockdown significantly decreased the levels of p-MTOR, p-Akt, p-Rps6, p-Erk1/2, p-Src while didn't alter the gross level of each kinase. (Fig. 6a and b). These indicated that the mechanisms that KBTBD8 regulates different kinases have different internal details.

Since KBTBD8 is an ubiquitin ligase and interacted with cul3 in neurons (Werner et al. 2015), we also verified that KBTBD8 interacted with cul3 (Fig. 6c). Moreover, we also verified that KBTBD8 knockdown significantly reduced the overall protein ubiquitination of ovarian cancer cells (Fig. 6d and e, Additional file 3: Fig. 4). These results implied that a high KBTBD8 level is important for the activation of multiple important tumorigenesis-related kinases, probably through ubiquitinating and maintaining their kinase activities.

### KBTBD8 knockdown efficiently minimize tumor growth in vivo

**Fig. 7:**
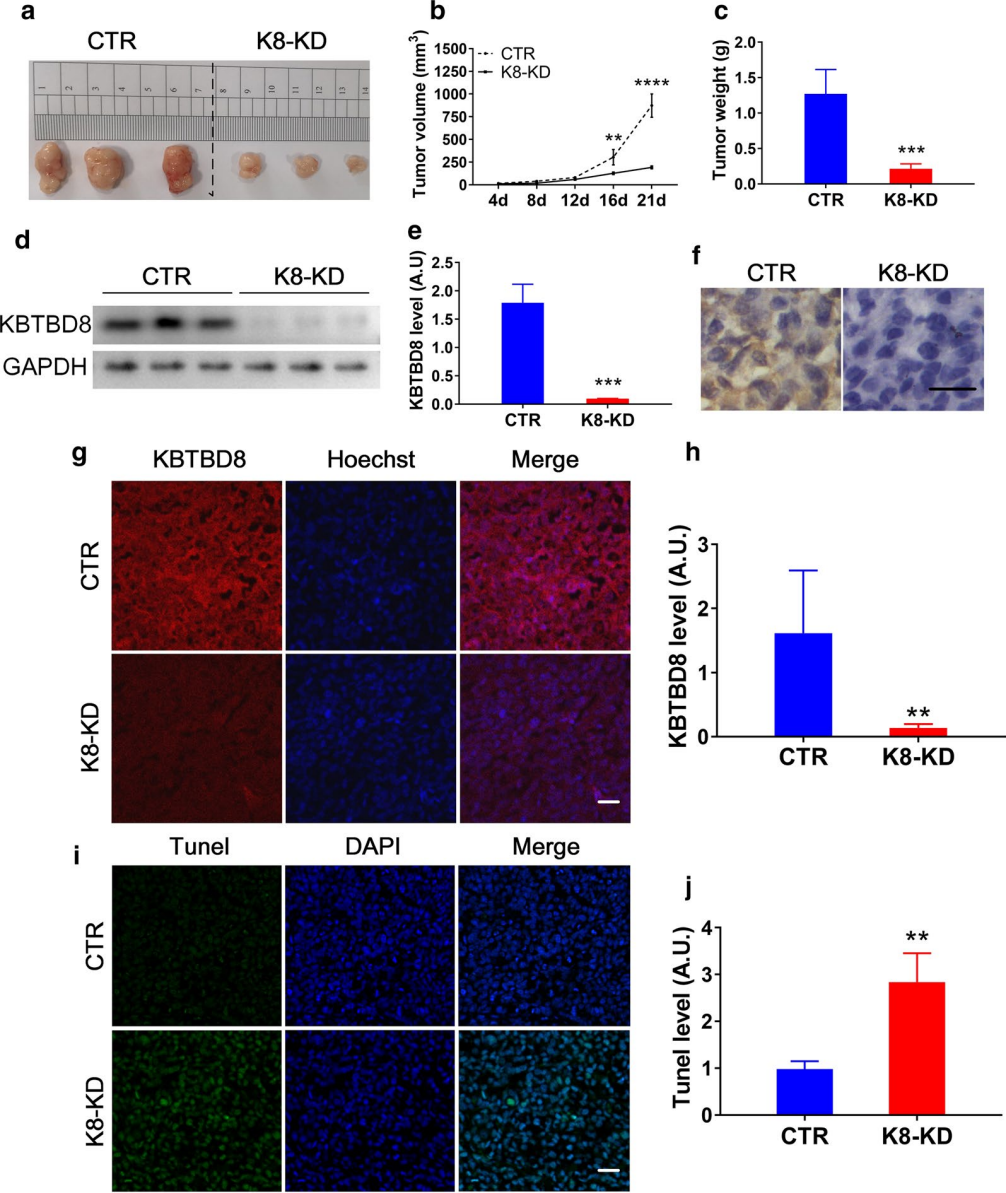
KBTBD8 knockdown suppressed tumor formation in vivo in A2780 cell xenograft model. **a**–**c** In vivo tumor formation assay in A2780 cell xenograft model. Both tumor volume (tumor volume at 21d, CTR vs. K8-KD, 872.7mm^3^ vs. 191.7 mm^3^) and tumor weight (tumor weight at 21d, CTR vs. K8-KD, 1.270 g vs. 0.2142 g) were significantly reduced in the KBTBD8-knockdown A2780 cell xenograft group. **d**, **e** Western blot and quantification showed that the KBTBD8 protein level was still significantly lower (KBTBD8 level, CTR vs. K8-KD, 1.790 vs. 0.09559) in the KBTBD8-knockdown group at the examination time point. **f** Immunohistochemistry of tumor tissues verified that KBTBD8 signal was still significantly lower in the KBTBD8-knockdown group at the examination time point (Three weeks after the xenograft). **g**, **h** Immunofluorescence also showed that KBTBD8 signal was still significantly lower (KBTBD8 signal, CTR vs. K8-KD, 1.619 vs. 0.1354) in the KBTBD8-knockdown group at the examination time. KBTBD8 in red, DNA in blue. **i**, **j** Tunel assay showed that apoptosis signal significantly increased in KBTBD8-knockdown tumor than in control (Tunel level, CTR vs. K8-KD, 0.9770 vs. 2.840). Tunel signal in green, DNA in blue. Scale bar, 50 μm. **Indicates p < 0.01, ***indicates p < 0.001, ****indicates p < 0.0001

Finally, we analyzed the effects of KBTBD8 in vivo. We subcutaneously transplanted a prescribed number of control or K8-KD EOC cells into immunodeficient nude mice and checked the tumor formation after three weeks. We found that both tumor size and weight were significantly reduced by more than threefold in the KB-KD A2780 (Fig. 7a–c) or HO8910 cell xenograft group (Additional file 3: Fig. 5a–c). A parallel blot of each tumor at the examination time showed that the KBTBD8 level between tumors inside control or K8-KD group was very identical (Fig. 7d and e), and KBTBD8 levels in K8-KD group were all significantly lower than in control tumors (Fig. 7d and e, Additional file 3: Fig. 4E and 5D). Immunohistochemistry and immunofluorescence also showed that KBTBD8 signal was still significantly lower in the K8-KD group at the examination time (Fig. 7f–h, Additional file 3: Fig. 5F–H). And furthermore, Tunel assay showed that the apoptotic signal was significantly higher in the KBTBD8-knockdown tumors than in the control tumors (Fig. 7i and j, Additional file 3: Fig. 4J and 5I).

These results indicate that KBTBD8 knockdown impedes ovarian tumor progression in vivo as effectively as when evaluated in vitro. We have to note that nude mice, although widely used in cancer studies, are immunodeficient and may not fully reflect the actual immunity situation of the host, but the sharp decrease in tumor growth appears to be the effect of KBTBD8 knockdown.

## Discussion

In the present study, we found for the first time that KBTBD8 was significantly upregulated in various clinical human ovarian cancer tissues and cultured cell lines. KBTBD8 knockdown significantly reduced tumor formation in nude mice. In addition, KBTBD8 regulate multiple tumorigenesis-related genes and kinases.

Although KBTBD family members are not known to be involved in EOC (nor in other types of cancer) progression, proteins with kelch or BTB homologous motifs appear to be involved. For example, the overexpression of kelch-like ECH-associated protein 1 (KEAP1) significantly enhances cisplatin sensitivity (van Jaarsveld et al. 2013), and the downregulation of KEAP1 is associated with NF-E2-related factor 2 (Nrf2) pathway activation and induced cisplatin resistance (Konstantinopoulos et al. 2011). Regarding the BTB/POZ domain motif, nucleus accumbens-1 (Nac1 or NAC-1) belongs to the BTB/POZ gene family and is significantly overexpressed in ovarian serous carcinomas, and the induced expression of the NAC-1 mutant containing only the BTB/POZ domain disrupts NAC-1 bodies, prevents tumor formation, and promotes tumor cell apoptosis (Nakayama et al. 2006). NAC-1 knockdown also increases paclitaxel sensitivity (Jinawath et al. 2009). KBTBD8 could therefore potentially function in EOC progression in a fashion similar to both kelch and BTB family proteins, and this portends broader impacts. Actually kelch or BTB-containing proteins have been shown to regulate the progression of other type of tumors. For example, 20% of KRAS (Kirsten rat sarcoma viral oncogene)-mutant LAUD (lung adenocarcinoma) human tumors carry loss-of-function mutation in keap1. In KRAS-driven LAUD model mice, keap1 deficiency caused NRF2 overactivation and glutaminolysis increment, thereby promoted tumor progression (Romero et al. 2017). Increased lipid synthesis is a main character of many cancers, whereas ATP-citrate lyase (ACLY) is a key enzyme for lipid synthesis and is frequently overexpressed or activated in cancer to promote tumor progression. A Kelch-like family member, KLHL25, bound cul3 to ubiquitinate and degrade ACLY, thereby inhibit inhibits tumor progression of lung cancer cells (Zhang et al. 2016). EMT (Epithelial–mesenchymal transition) is a critical step in the metastasis of hepatocellular carcinoma (HCC). A BTB/POZ family member, BTBD7, was highly expressed in HCC cells and tumor and was associated with enhanced cell motility, venous invasion, and poor prognosis. Increased BTBD7 activated RhoC-Rock2-FAK-signaling pathway and conduced to MMP (matrix metalloproteinase)-2/9 production and microvessel formation (Tao et al. 2013). KBTBD8 contains both kelch and BTB domains, thus presumably its overexpression & overactivation could be more important in EOC progression.

Various ubiquitination complexes have many substrates, and it is therefore possible that KBTBD8 regulates multiple essential proteins. The comparative RNA sequencing revealed many important differentially expressed genes that were validated and might therefore be involved in EOC progression. For example, among the upregulated genes, H19 opposite tumor suppressor (HOTS) is encoded by a human imprinted H19 antisense transcript and is localized to the nucleus and nucleolus. HOTS overexpression inhibits the growth of multiple types of cancer, while HOTS knockdown increases tumor progression in vitro, in accordance with sequencing results where KBTBD8 knockdown caused HOTS upregulation (Onyango and Feinberg 2011). Among the downregulated genes, CXC motif chemokine receptor 4 (CXCR4) encodes a CXC chemokine receptor specific for stromal cell-derived factor-1, and the inhibition of either CXCR4 or its ligand, CXCL12, reduces tumor growth in vitro and prolongs the survival of immunocompetent mice infused with ovarian cancer cells (Darash-Yahana et al. 2004; Righi et al. 2011). These effects agree with the sequencing results, where KBTBD8 knockdown caused CXCR4 downregulation. Actually, kelch or BTB-containing proteins have also been shown to have plenty of potential substrates through transcriptome studies. For example, in lung adenocarcinoma (LAC) cohorts, patients who carried mutations in the KEAP1/NFE2L2 pathway had significantly shorter survival. These patients had elevated expression of ATM (ataxia telangiectasia mutated) and ATR (ataxia telangiectasia and Rad3-related), which are two important kinases involved in tumorigenesis (Goeman et al. 2019). ZBTB28/BCL6B/BAZF is a BTB/POZ domain protein methylated in multiple tumors. ZBTB28 expression inhibits multiple oncogenic signaling including NF-κB, MAPK, wnt, VEGF, and JAK-STAT in tumor cells (Xiang et al. 2019). Mutation in BTB and CNC homology 1 (BACH1) has been associated with pancreatic ductal adenocarcinoma (PDAC) risk. BACH1 knockdown of promoted cell proliferation and angiogenesis in PDAC. Gene ontology analysis showed that BACH1 knockdown upregulated genes in angiogenesis, MTORC1, hypoxia, MYC targets, P53, and epithelial-mesenchymal transition (Huang et al. 2018). Since Kelch or BTB family proteins have been shown in multiple tumorigenesis-related pathways, and coupled with our RNA sequencing results, KBTBD8 appeared to function in multiple tumorigenesis-related pathways as well.

KBTBD8 is the only member of the KBTBD family identified as a female fertility factor (Gallardo et al. 2007), and we showed that KBTBD8 inhibition prevented ovarian cancer progression; this finding suggests that KBTBD8 is an essential protein for both normal female fertility and for the normal growth of ovarian somatic cells. We refer to this phenomenon as the "dual function" of KBTBD8, and some crucial proteins are known to function similarly. For example, BRCA1 mutations are well-known to be frequently linked with the occurrence of human ovarian cancer (Titus et al. 2013; Hoskins and Gotlieb 2017; George et al. 2017; Li et al. 2016). BRCA1 is also required for DNA double-strand break (DSB) repair, and a BRCA1 deficiency in female mice results in increased oocyte apoptosis, which favors decreased primordial follicle reserve and impaired fertility (Hoskins and Gotlieb 2017). Similarly, in women, BRCA mutations increase oocyte DNA damage and reduce primordial follicle reserve (Hoskins and Gotlieb 2017). Furthermore, we discovered that KBTBD8 knockdown inactivated multiple essential kinases. Gene mutation or amplification of mTOR, Erk, Akt, or β-catenin has been frequently characterized in human high-grade ovarian cancer and is positively correlated with drug resistance (Caumanns et al. 2018; Rahman et al. 2013; Davies et al. 2015; Wu et al. 2001; Palacios et al. 1998), while the downregulation of any of these genes appears to inhibit ovarian cancer progression and promote drug sensitization (Pétigny-Lechartier et al. 2017; Lu et al. 2014; Au-Yeung et al. 2017; Chartier et al. 2016). As these kinases have also been shown to be important for female fertility, KBTBD8 is thus important not only for the normal function of ovarian epithelial cells, but also for the normal fertility.

## Conclusions

In conclusion, we demonstrated for the first time that the female fertility factor KBTBD8 is also essential for the normal functioning of ovarian epithelial cells. KBTBD8 overexpression is positively correlated with the malignancy grade of ovarian cancer. KBTBD8 knockdown greatly reduced EOC progression both in vitro and in vivo. KBTBD8 may function via multiple essential proteins and signaling pathways.

## Supplementary information

**Additional file 1: Dataset 1.** This table includes scores of all the points of the ovarian tissue microarray. Table 1 in the results part was generalized from this table. **Dataset 2:** This table contains all upregulated genes and related information in the KBTBD8-knockdown group in contrast to control ($\left| {\log 2{\text{FC}}} \right| \ge 1$). **Dataset 3:** This table contains all downregulated genes and related information in the KBTBD8-knockdown group in contrast to control ($\left| {\log 2{\text{FC}}} \right| \ge 1$). **Dataset 4:** This table includes all 39 genes and related information that encoding proteins interacting with the KBTBD8-regulated multiple essential cell cycle kinases in Fig. 2l.

**Additional file 2: Table 1.** KBTBD8 shRNA information. This table shows all the sequences and locations of human KBTBD8 shRNAs used in this study. shRNAs were cloned into the plasmid GV248 cutted by AgeI/EcoRI. GV248 vector map is attached below. **Table 2**: Primers for Flag-6 × leu-KBTBD8-EGFP in pcDNA3.1 (+). This table includes primers for amplifying full-length CDS (coding sequences) of KBTBD8 and EGFP. pcDNA3.1 (+) vector map is attached below. **Table 3:** Primers for Q-PCR. This table outlines all primers for Q-PCR in Fig. 2h–j. **Table 4:** KEGG pathway classification of all differentially-expressed genes in KBTBD8-depleted cells. This table outlines all carcinogenesis-related differentially expressed genes in KBTBD8-depleted cells by KEGG pathway classification in Fig. 7k.

**Additional file 3: Figure 1.** Results in HO8910 showed that human KBTBD8 was important for the proliferation of ovarian epithelial cancer cells. **A and B** Blot and quantification showed that KBTBD8 protein level was about tenfold higher in HO8910 ovarian cancer cells than in normal cell line moody (KBTBD8 protein level, Moody vs. HO8910, 0.01478 vs. 0.1490). **C and D** Blot and quantification showed that KBTBD8 protein was significantly reduced by specific shRNA. **E and F** The number of colonies formed after 7 days was significantly decreased in KBTBD8-knockdown (K8-KD) compared with the numbers in control cells (colony numbers, CTR vs. K8-KD, 135 vs. 49). **G–I** Cell cycle analysis by FACS showed that significantly fewer cells were at S stage (proportion of cells at S phase, CTR vs. K8-KD, 45.88% vs. 21.24%) and considerably more cells were at the G1 stage (percentage of cells at the G1 phase, CTR vs. K8-KD, 48.37% vs. 69.90%) in KBTBD8-knockdown HO8910 cells than in control cells. **J** CCK8 assay also showed that there were significantly less proliferating cells in the KBTBD8-knockdown HO8910 cells than in control cells (CCK8, CTR vs. K8-KD, 0.8500 vs. 0.6259). A.U., arbitrary unit. *Indicates p < 0.05, ** indicates p < 0.01, *** indicates p < 0.001. **Figure 2:** Results in HO8910 cells showed that human KBTBD8 was important for the migration of EOC cells. **A and B** Would healing assays showed that the closure ratio of the scratched area was slower in the KBTBD8-knockdown HO8910 cells than in the control cells. Representative images at 0 h, 24 h, 48 h, and 72 h were shown. Percentage of initial area uncovered by migrated cells at 24 h, CTR vs. K8-KD, 56.03% vs. 86.67%; at 48 h, 20.34% vs. 66.68%; at 72 h, 8.719% vs. 55.31%. **C and D** 3-dimensional migration assay showed that the migrated cells were significantly less in the KBTBD8-knockdown than in the control cells (Number of migrated cells per field, CTR vs. K8-KD, 216 vs. 22). Scale bar, 50 μm. ****Indicates p < 0.0001. **Figure 3:** Results in HO8910 showed that human KBTBD8 was important for the general health state of ovarian epithelial cancer cells. **A and B** KBTBD8 knockdown significantly increased ROS level by eightfold in HO8910 ovarian cancer cells (ROS level, CTR vs. K8-KD, 1.508 vs. 12.26). **C and D** JC-1 staining assay showed that the mitochondria membrane potential significantly decreased by tenfold after the KBTBD8 knockdown (JC1 level, CTR vs. K8-KD, 1.390 vs. 0.1330). Aggregate in green, monomer in red. **E and F** Annexin V staining assay showed that KBTBD8 knockdown significantly increased the apoptosis level (Annexin V level, CTR vs. K8-KD, 1.857 vs. 5.738). Annexin V signal in green, PI in red. Scale bar, 50 μm. ***Indicates p < 0.001, **** indicates p < 0.0001. **Figure 4:** KBTBD8 knockdown decreased the overall ubiquitination level in A2780 EOC cells. A-C related to Fig. 6d and e, three repeats all showed that KBTBD8 knockdown decreased the overall ubiquitination level in A2780 EOC cells. **Figure 5:** KBTBD8 knockdown suppressed tumor formation in vivo in HO8910 cell xenograft model. **A–C** In vivo tumor formation assay in HO8910 cell xenograft model. Both tumor volume (tumor volume at 21d, CTR vs. K8-KD, 490.4 mm^3^ vs. 138.2 mm^3^) and weight (tumor weight at 21d, CTR vs. K8-KD, 0.5777 vs. 0.1451) were significantly reduced in the KBTBD8-knockdown HO8910 cell xenograft group. **D and E** Western blot and quantification showed that the KBTBD8 protein level was still significantly lower (KBTBD8 level, CTR vs. K8-KD, 0.1385 vs. 0.02099) in the KBTBD8-knockdown group at the examination time point. **F** Immunohistochemistry of tumor tissues verified that KBTBD8 signal was still significantly lower in the KBTBD8-knockdown group at the examination time point (Three weeks after the xenograft). **G and H** Immunofluorescence also showed that KBTBD8 signal was still significantly lower (KBTBD8 signal, CTR vs. K8-KD, 1.033 vs. 0.01459) in the KBTBD8-knockdown group at the examination time. KBTBD8 in red, DNA in blue. **I and J** Tunel assay showed that apoptosis signal significantly increased in KBTBD8-knockdown tumor than in control (Tunel level, CTR vs. K8-KD, 1.117 vs. 1.539). Tunel signal in green, DNA in blue. Scale bar, 50 μm. **Indicates p < 0.01, *** indicates p < 0.001, **** indicates p < 0.0001.

## Data Availability

The data that support the findings of this study are available from the corresponding author upon reasonable request.

## Abbreviations

KBTBD8: Kelch repeat and BTB domain-containing protein 8
IP: Immunoprecipitate
ROS: Reactive oxygen species
PKM2: Pyruvate kinase isoenzyme M2
KEAP1: Kelch-like ECH-associated protein 1
Nrf2: NF-E2-related factor 2
Nac1: Nucleus accumbens-1
BRCA1: Breast cancer type 1
DSB: DNA double-strand break
HOTS: H19 opposite tumor suppressor
CXCR4: C-X-C motif chemokine receptor 4
EOC: Epithelial ovarian cancer
SMARCAD1: SWI/SNF-related, matrix-associated actin-dependent regulator of chromatin, subfamily a, containing DEAD/H box 1
KRAS: Kirsten rat sarcoma viral oncogene
LAUD: Lung adenocarcinoma
ACLY: ATP-citrate lyase
EMT: Epithelial-mesenchymal transition
HCC: Hepatocellular carcinoma
BACH1: BTB and CNC homology 1
PDAC: Pancreatic ductal adenocarcinoma
